# Effect of Magnetite Concrete on Splitting Tensile Strength and Gamma Ray Shielding Performance Exposed to Repeated Heating at High Temperature

**DOI:** 10.3390/ma16072592

**Published:** 2023-03-24

**Authors:** Xinyun Huang, Zhenfu Chen, Qiuwang Tao, Liping Xie, Dan Jin, Dan Wu

**Affiliations:** 1 School of Civil Engineering, University of South China, Hengyang 421001, China; 2 Key Laboratory of High-Performance Special Concrete in Hunan Province, University of South China, Hengyang 421001, China; 3 Key Laboratory of High-Performance Concrete in China Nuclear Industry, University of South China, Hengyang 421001, China; 4 China Nuclear Industry 22ND Construction Company Ltd., Yichang 443101, China

**Keywords:** high temperatures, multiple heating, magnetite shielded concrete, splitting tensile strength, gamma-ray shielding properties

## Abstract

Radiation shielding concrete is one of the most used materials in the construction of nuclear power plants and will be subjected to high temperatures for a long time during its service life. This study aims to investigate deterioration of radiation shielding concrete with multiple heating at different temperatures. A microwave oven was used as a heating apparatus to simulate irradiation, and 200, 300, and 400 °C were selected as experimental cycle temperatures. The apparent characteristics, mass loss, splitting tensile strength, and gamma ray shielding properties of the commonly used magnetite shielding concrete were investigated. The results showed that the splitting tensile strength and gamma shielding performance of concrete were dramatically reduced at first heating. Then, as the heating times increased, the splitting tensile strength and gamma shielding properties of the concrete continued to deteriorate, and the higher the increase in heating temperature, the more severe the deterioration of the concrete. During the service period of radiation shielded concrete, the magnitude of temperature under the service conditions will affect the deterioration degree of concrete, and the continuous change of temperature will continuously lead to the deterioration of concrete.

## 1. Introduction

Concrete is one of the most common and inexpensive materials used in facilities that contain radioactive sources and radiation generating equipment. In contrast to other construction materials, concrete has better shielding properties and good durability against nuclear radiation [1,2,3]. Radiation shielding concrete, also known as heavy concrete, is a special type of concrete. By using high-density materials (such as magnetite [4,5,6,7,8]) as aggregates or adding various high-density admixtures, and introducing sufficient amounts of crystalline water or light element compounds, the shielding properties of concrete can be better suited to different circumstances [9,10,11]. Compared with ordinary concrete, radiation shielding concrete, with better shielding performance, can effectively attenuate gamma radiation, neutron radiation, and both mixed fields [12,13,14]. Therefore, it is widely used in major defense projects, including air defense projects, military security infrastructure, nuclear reactor containers, buildings for storage, and disposal of nuclear waste.

In general, radiation shielding concrete is exposed to high temperature and high radiation environment for a long time. The temperature of radiation shielding concrete in nuclear power plant is between 60 °C and 120 °C under normal working conditions [15]. In nuclear reactors, the absorption of radiation energy can raise the temperature of concrete to 250 °C, and the temperature of thicker radiation-shielding prestressed concrete can reach 400 °C. Additionally, in the service life, radiation shielding concrete suffers from force, heat, or combined effects, which cause serious deterioration in its mechanical properties and radiation shielding performance [2,16,17]. Therefore, understanding thermal distribution, thermal effects, and thermal stress on radiation shielding concrete has become a significant aspect affecting its design [17,18,19].

High temperature is an essential indicator of the deterioration of the performance of radiation shielding concrete. Due to the poor thermal conductivity, the concrete is subject to internal temperature differences under the direct action of the reactor core, which ultimately causes concrete cracking and a decline in durability [20]. Nowadays, the main theories concerning thermal damage of concrete are thermal stress mechanism and steam pressure mechanism. The temperature stress mechanism indicates that thermal stresses will be generated internally under the effect of temperature because concrete is a poor conductor of thermal, and the greater the effect of thermal stresses as the temperature increases. When the thermal stress surpasses the tensile strength of concrete, the concrete will fracture [21,22]. The steam pressure mechanism claims that when the internal steam pressure is high enough, it will lead to concrete cracking [23]. The above two theories demonstrate that the damage inside the concrete depends on internal thermal stress or vapor pressure and the ability of the concrete to withstand it [24]. Therefore, the tensile performance of concrete is the main factors that determine the brittle damage of concrete, and also directly affect the cracking and durability of concrete structures [25].

The thermal damage of concrete at high temperatures mainly consists of structural cracking (physical effect) and hydride decomposition (chemical effect). There are at least 3 kinds of cracks in concrete at high temperatures including shrinkage cracking induced by hydride decomposition [26], by thermodynamic mismatch between aggregates and cement paste [26,27] and by temperature gradients. In the rapid rising temperature process, the number of cracks and pore water (steam) pressure will increase, thus constantly generating new cracks, resulting in the decrease in concrete strength [28]. In general, the hydride compounds of concrete will keep decomposing with temperature climbing, which lead to the continuous deterioration of concrete performance [29,30,31]. The free water in the concrete pores is vaporized at 105 °C. The calcium alumina in the concrete is decomposed at 80–200 °C [31]. The crystalline water in the cementitious material will be degraded at 300 °C. At 450 °C, calcium hydroxide, a hydration product of cement, will start to be decomposed, and at 600 °C, it will be completely decomposed [32]. Meanwhile, because the mass of aggregates in heavy concrete will account for most of the total mass, they play a key role in the fire resistance of concrete. Magnetite mainly contains ferric tetroxide, which has good thermal stability. Consequently, the magnetite aggregates maintain good phase stability at 570 °C [33,34]. At present, the research on radiation shielding concrete is primarily focused on normal temperature [35,36,37,38,39,40,41], and there is less research on high temperature. Nevertheless, some scholars have also started to study the effects of high temperature on the mechanical performance, shielding properties, and microstructure in radiation shielding concrete owing to the existence of high temperature effects during the service period of radiation shielding concrete [42,43,44,45].

The patterns of apparent properties, mass loss, splitting tensile strength, and gamma shielding properties of magnetite radiation shielding concrete heated at 200, 300, and 400 °C for different times were investigated in this paper. It explores the deterioration of radiation shielding concrete at different temperatures with multiple heating cycles, which provides some reference significance for the application of radiation shielding concrete.

## 2. Materials and Methods

In this section, the basic properties of the material, the preparation of the specimen, and the main experimental methods will be introduced.

### 2.1. Materials

The test used P.O 42.5 ordinary silicate cement from Hengyang City, Hunan Province, whose chemical composition is shown in Table 1.

**Figure 1:**
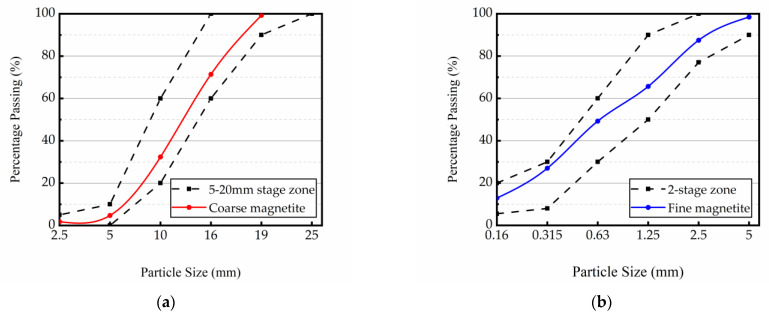
(**a**) Grading curve of magnetite coarse aggregates; (**b**) Grading curve of magnetite fine aggregates.

The magnetite ore came from Gongyi City, Henan Province. Reference JGJ 52-2006 for test measures and standards. After cleaning and drying, the raw stones were screened using a standard square-hole sieve with needle and flakes removed, and then placed in different containers according to different particle sizes. The coarse aggregates used in the tests had a maximum particle size of 20 mm and were mixed in accordance with a certain mass ratio in below 5 mm, 5–10 mm, 10–16 mm, and 16–20 mm sieve size range. Part of 5–20 mm coarse aggregate was obtained by crushing coarse aggregate larger than 20 mm and part of 0.16–1.25 mm fine aggregate was obtained by crushing fine aggregate of 1–5 mm. The chemical composition of magnetite aggregates is illustrated in Table 1. The basic properties and gradation curves of magnetite coarse and fine aggregates are shown in Table 2 and Figure 1.

### 2.2. Mixing Proportion and Samples Preparation

The test required magnetite radiation shielding concrete. The initial mix proportion was calculated according to the iron ore radiation shielding concrete mix ratio calculation method specified in GB/T 34008-2017. The mix ratio of magnetite concrete is shown in Table 3.

According to GB/T 5001-2019 standard, concrete specimens were made with the size of 100 mm × 100 mm × 100 mm. The test specimens were demolded after one day of resting after pouring, and then put into the standard curing room for 28 d. After the test specimens were cured, the specimens were placed in a dry atmospheric area for 14 d.

### 2.3. Test Methods

#### 2.3.1. Test of High Temperature Cyclic Heating

**Figure 2:**
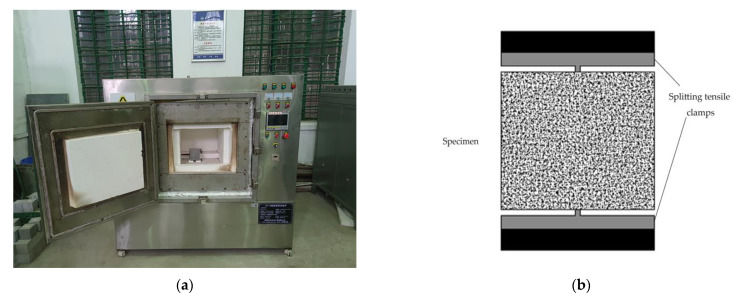
(**a**) Heating diagram; (**b**) Schematic diagram of splitting tensile test.

All concrete specimens were heated sequentially in a microwave oven (Simulated radiation environment—internal heating) with an internal chamber size of 400 mm × 480 mm × 340 mm. The same loading power was used for each heating (6 microwave emitters) and the temperature was measured at the center of the left side of the concrete (the specimen will be marked on the front side) to ensure the consistency of each loading (Figure 2). We placed only one specimen per heating in the oven, and tried to ensure that the placement position remains unchanged to ensure the consistency of the microwave field and heating trend to which the heating was subjected. Then, the bottom of the specimen was padded with non-wave absorbing and high temperature resistant material to allow for heat exchange at the bottom of the specimen. This test set 6 kw to heat the concrete sample. When the temperature of specimen, locating at lateral center point reaches the target temperature (200 °C, 300 °C, 400 °C), the heating process was stopped. The specimen was placed in the air to cool down to room temperature. Then, we repeated the above test steps until the specimen was heated to the target number of times (1, 3, 5, 10). Subsequently, stopped the test, and wrapped the specimen in preservative film.

#### 2.3.2. Mechanical Tests

The 10 cm × 10 cm × 10 cm concrete specimens were tested for splitting tensile strength, and the specimens conformed to Chinese standard GB/T5001-2019. We performed splitting tensile tests on the specimens using WAW-EY600C electro-hydraulic pressure servo tester with load control and a load rate set at 0.05 MPa/s (Figure 2). Three magnetite concrete specimens were tested at each cycle and the mean of their measurements were taken as results.

#### 2.3.3. Gamma Radiation Shielding Test

**Figure 3:**
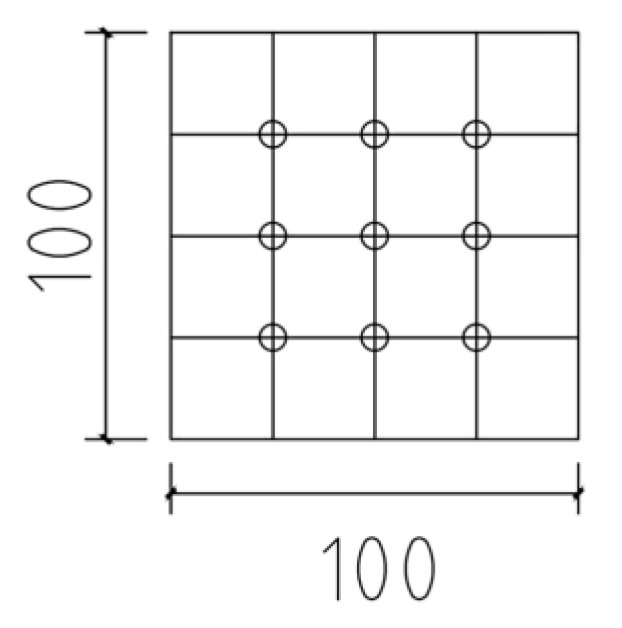
Distribution of nine test points.

**Figure 4:**
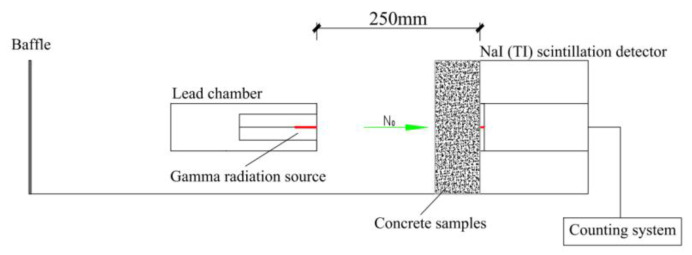
Gamma radiation shielding test diagram.

Gamma radiation shielding tests on magnetite concrete were made in the BH1326 nuclear physics experiment platform with NaI (TI) scintillation detector, a counting system and a gamma radiation source. The 137Cs radioisotope was used as a gamma-ray source which has 0.662 MeV photon energy (the source of 137Cs was a cylinder with a diameter of 5 mm and a height of 1 cm. Its activity was about 10 millicurie). The 10 cm × 10 cm × 10 cm specimens was cut into test specimens with thicknesses of 2.5 cm, 5.0 cm, and 7.5 cm, respectively. The specimens used for the test with a thickness of 10 cm were spliced from 2.5 cm and 7.5 cm specimens. To reduce thickness inhomogeneities and differences in aggregate distribution, each concrete sample was repeatedly tested at nine test points (Figure 3). Under gamma irradiation lasting 60 s, each experimental point was measured, and the average of 9 points was used as a result. Figure 4 depicts the device schematic of the gamma shielding test.

In this experiment the linear attenuation coefficient (μ), half-value layer (HVL), ten-value layer (TVL), mean free path (Mfp), and mass attenuation coefficient (Mac) of test specimen was discussed. Equations (1)–(5) is to calculate the μ, HVL, TVL, Mfp, and Mac of concrete, respectively. Equation (6) is the error calculation formula of linear attenuation coefficient [5]. According to the error transfer equation, the error of HVL, TVL, Mfp, and Mac can be calculated, respectively. When it decays to the original value of radiation intensity attenuation for 50% and 10%, the required concrete thickness values are called HVL and TVL, respectively. They can be used for identifying the gamma shielding capacity of material [38].

I = I_0_ × e^−μx^ (1)

HVL = ln2/μ(2)

TVL = ln10/μ(3)

Mfp = 1/μ(4)

μ_m_ = μ/ρ (5)

∆μ = 1/x × {(∆I_0_/I_0_)^2^ + (∆I/I)^2^ + [ln(I_0_/I)^2^] × [∆x/x]^2^} (6)

I is the gamma ray count rates (excluding the background count rates) through a test specimen of thickness x; I_0_ is the count rates of blank control; x means the thickness of the test specimen (cm); μ is the linear attenuation coefficient of test sample (cm^−1^); μ_m_ refers to the mass attenuation coefficient of the test sample with gamma rays(cm^2^/g); ρ represents the density of the test specimen (g/cm^3^); the error of μ, I, I_0_, μ_m_ and x are Δμ, ΔI, ΔI_0_, Δμ_m,_ and Δx, respectively.

## 3. Results and Discussion

In this section, the deterioration of splitting strength with different number of heating at different cyclic temperatures and the effect of different number of heating at different cyclic temperatures on the linear attenuation coefficient and transmission rate are presented.

### 3.1. Apparent Characteristics

**Figure 5:**
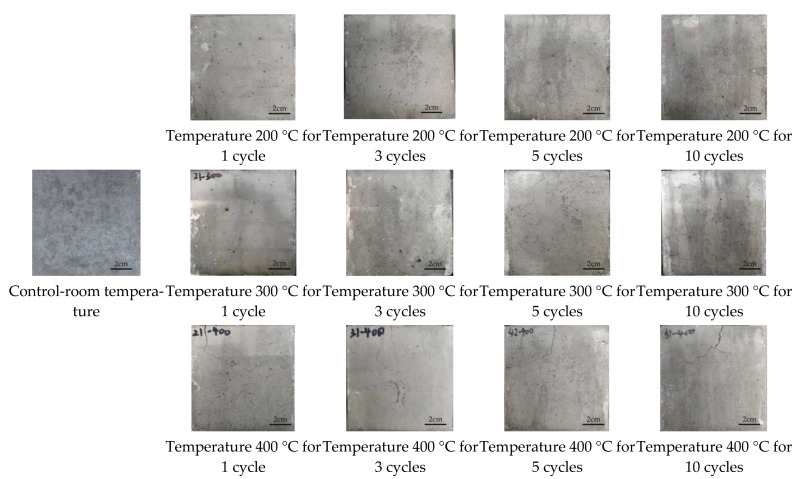
Apparent characteristics of magnetite concrete at high temperature with repeated heating.

The appearances of magnetite concrete at high temperature with repeated heating are shown in Figure 5. The results showed that with the increase in heating temperature, the colors of the concrete specimen surface were gray-blue, gray, light gray, and gray-white, and the increase in heating times had no significant effect on the surface color. The fine cracks appeared in the specimens at the first heating at 200 °C, and the length and width of the cracks increased with the repeated heating. At the heating temperature of 200 °C and 300 °C, the cracks were not obvious; however, after 10 times of repeated heating, more obvious cracks appeared above the specimen. The visible cracks could be seen at the first heating of the specimen at 400 °C. The magnetite concrete maintained excellent integrity in the experiments owing to the high contents of ferric tetroxide in the magnetite aggregate, which is stable in high temperatures and has good thermal conductivity and high temperature thermal storage properties.

### 3.2. Mass Lost

**Figure 6:**
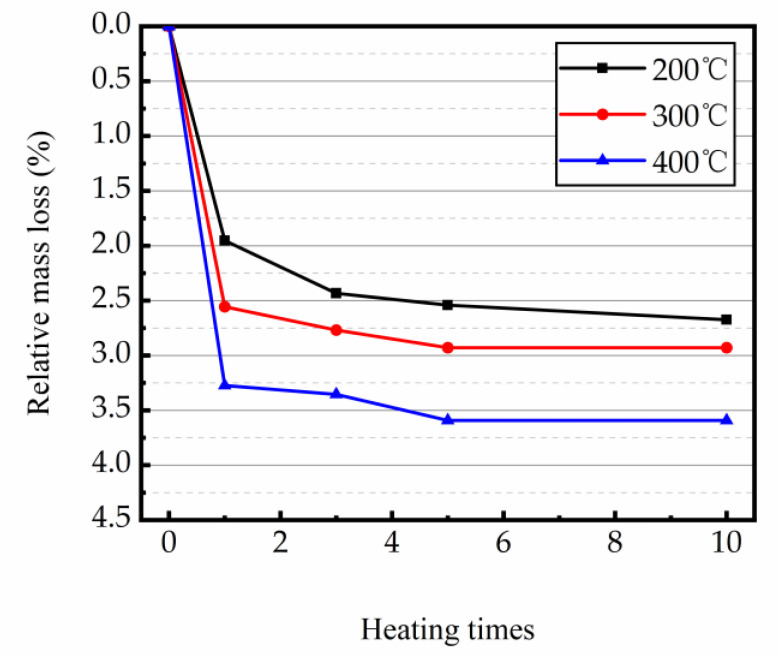
Relative mass loss of magnetite concrete at high temperature with repeated heating.

The concrete internal composition changes with the temperature increasing, which will result in variations in the quality of the test samples. Thus, the change in the quality of concrete repeatedly heated at high temperatures can be used to indirectly assess the degree of deterioration and the status of changes in the internal composition of concrete. Figure 6 represents the relative mass loss of magnetite concrete at high temperature with repeated heating.

The experimental results showed that the magnetite samples changed the most in relative mass loss at the first round of heating, and the higher the heating temperature, the greater the relative mass loss. With the increase in heating times, the relative mass loss of the magnetite samples at heating temperature of 200 °C gradually increased and the trend of change decreased. While the relative mass loss of magnetite samples heated at 300 °C and 400 °C tends to level off with the increase in heating times to a certain value. As the mass loss of magnetite concrete is largely due to the dissipation of water inside it and the decomposition of hydration products to a certain extent [29,30,31]. Furthermore, the degree of water dissipation and hydride decomposition varied with increasing temperature [46]. When heated for the first time, most of the free water and some hydrates within the specimen evaporated and decomposed, which resulted in a sharp increase in the relative mass loss of the specimen. The higher the temperature, the more complete the decomposition of cement matrix, and the larger the relative mass loss of specimen. With the increase in heating times, the relative mass loss of specimens heated at 300 °C and 400 °C tended to be constant at the temperature that the substances that can be evaporated and decomposed internally are consumed in more than one heating cycle. However, with the change in the heating times, the mass loss of test samples at the heating temperature of 200 °C was still gradually increasing, probably because the heating temperature was not too high. After 10 times of heating, the substances that can still decompose and evaporate at that temperature have not been completely decomposed.

Magnetite aggregates have excellent physical and chemical properties and good performance in thermal conductivity and thermal storage properties [32,33,47,48,49], thus the magnetite concrete specimens remained intact under repeated heating at high temperatures. The final relative mass loss changes were slight. In the case of specimens without shedding, it was seen that the major factor for the different final mass loss of specimens in the heating cycle loading of 200, 300, and 400 °C was the temperature difference, while the heating times was the process of reaching the final mass loss of specimens.

### 3.3. Splitting Tensile Strength Test

#### 3.3.1. Failure Features

**Figure 7:**
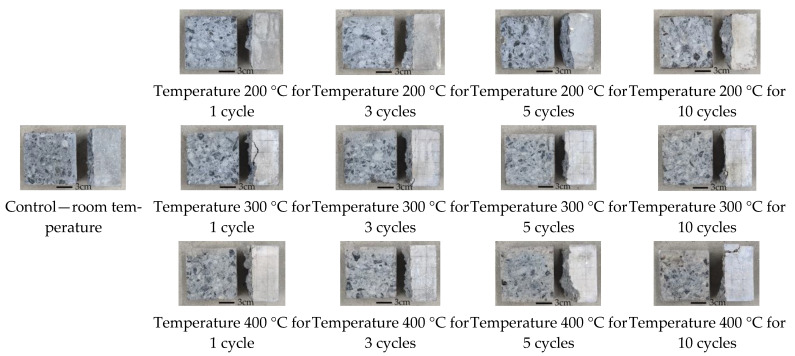
Splitting section characteristics of magnetite concrete at high temperature with repeated heating.

The features of the magnetite splitting blocks repeatedly heated at high temperatures are shown in Figure 7. The experimental results demonstrated that the internal color of the test samples at a heating temperature of 200 °C gradually changed from grayish-blue to light gray with increasing the number of heating times. The internal color of test samples at 300 °C changed directly from grayish green to light gray, and gradually changed from light gray to gray with increasing the number of heating times. The internal color of specimens at 400 °C changed directly from grayish green to gray, and gradually changed from gray to dark gray with increasing the times of heating. The fracture damage morphology of all three was changed from the interface damage morphology shared by mortar and aggregate to the damage morphology of the junction interface of aggregate-mortar first damage. To be specific, with the increase in the number of heating times and in heating temperature, the case of aggregate fragmentation at the damage surface was reduced and the damage of the junction interface of aggregate-mortar appears to increase.

Damage morphological transformation of concrete samples has three main reasons. One is the mortar and aggregates decompose differently at distinct temperatures. The decomposition degree of aggregates and mortar will keep decomposing with the increase in temperature [29,30,31,32]. The second is that aggregates and mortars have different thermal properties, so the thermal deformation of them at high temperatures is not coordinated. With repeated heating, they lead to the continuous formation and developing of cracks at the aggregate-mortar interface [26,27]. The third is based on thermal-stress mechanism and steam-pressure mechanism [24], the vapor pressure from water heating and the thermal stress from temperature changes acting inside the specimen can deteriorate the performance of the concrete.

#### 3.3.2. Splitting Tensile Strength

Table 4 shows the splitting tensile strength (f_t_, _T_) of magnetite concrete samples at high temperature condition. The experimental results indicated that as the heating time and temperature increased, the splitting tensile strength of magnetite concrete decreased continuously.

**Figure 8:**
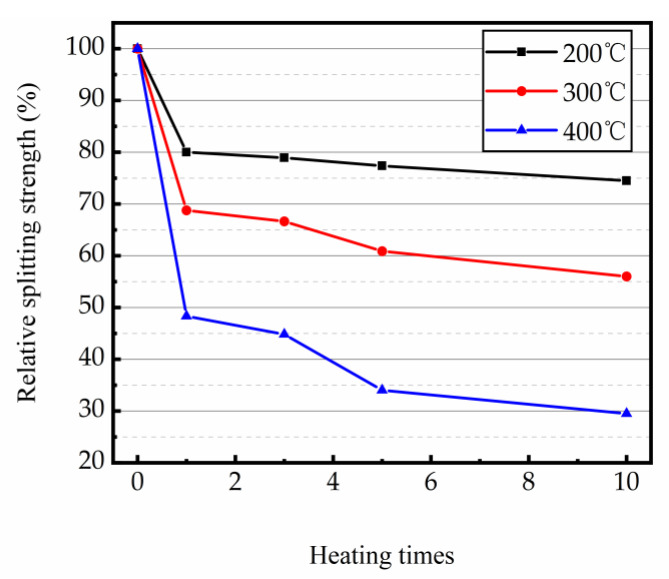
Relative splitting strength of magnetite concrete at high temperature with repeated heating.

Figure 8 shows the relative splitting intensity of the test samples at different temperatures and different heating times. The results demonstrated that as the number of heating times increased, the relative splitting strength of magnetite concrete specimen declined. The relative splitting strength of test sample was inversely proportional to the heating temperature. After the first heating, the concrete showed the greatest change in splitting strength, with the relative splitting strength of the specimens with the heating temperatures of 200, 300, and 400 °C being 80.01%, 68.77%, and 48.34% of the original samples, respectively. The relative tensile strength changes of samples at heating temperatures of 200, 300, and 400 °C were 5.52%, 12.77%, and 18.84% in the heating times of 1–10, respectively. The greatest change in the decrease in splitting strength was observed between the third and fifth heating. When the splitting strength of specimens were heated at 200, 300, and 400 °C, the relative splitting strength descended 1.57%, 5.77%, and 10.79%, respectively.

Combined with the changes in mass loss of the samples under the experiment and thermal decomposition law of the concrete cement matrix, thermal damage under repeated heating at high temperature can be classified into the following two periods. One is the period of combined damage of various factors within the concrete (major damage) [21,22,23,50]. The second is the period of fatigue damage (lasting effects) caused by thermal stress—thermal expansion (non-uniform changes in aggregates and mortar [50]) under uneven heating caused by temperature changes. From the conclusion of the test, it was clear that the splitting tensile strength of the specimen decreased sharply under the first heating. These were affected by a multiplicity of factors, including steam pressure generated by the free and bonded water inside concrete, the thermal stress caused by temperature changes and the deterioration of the concrete due to the decomposition of some hydrides. All combined to cause a dramatic reduce in the splitting tensile strength of concrete. Moreover, the damage of concrete by these three factors tends to increase with the increase in temperature, so the higher the temperature, the more serious the deterioration of mechanical performance of concrete. With the free and bound water inside the concrete dissipating and the internal hydrates that can decompose at that temperature completely disappearing, the change in the mechanical performance of concrete will transform from the first period to the second. It means that the thermal damage of concrete shifted from the period of combined damage by a variety of factors to the period of temperature change induced by the fatigue effect of thermal stress—thermal expansion which causes a continuous decrease in the ultimate tensile strength of specimen (300 °C and 400 °C in the second period after five times). The splitting strength of the specimens declined the fastest between 1 and 10 times of heating. Additionally, the higher the temperature, the more obvious this trend is. It is probably because the concrete internal cracks gradually connected under repeated heating, which caused a further decrease in the splitting tensile strength of concrete.

### 3.4. Gamma Radiation Shielding Performance

#### 3.4.1. The Linear Attenuation Properties

**Figure 9:**
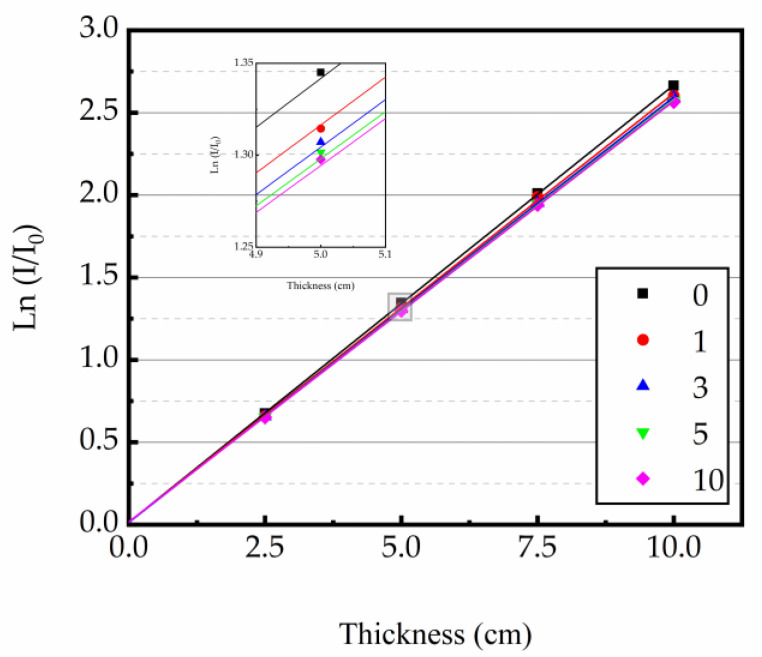
Relationship between ln(I/I_0_) and concrete thickness at 200 °C.

**Figure 10:**
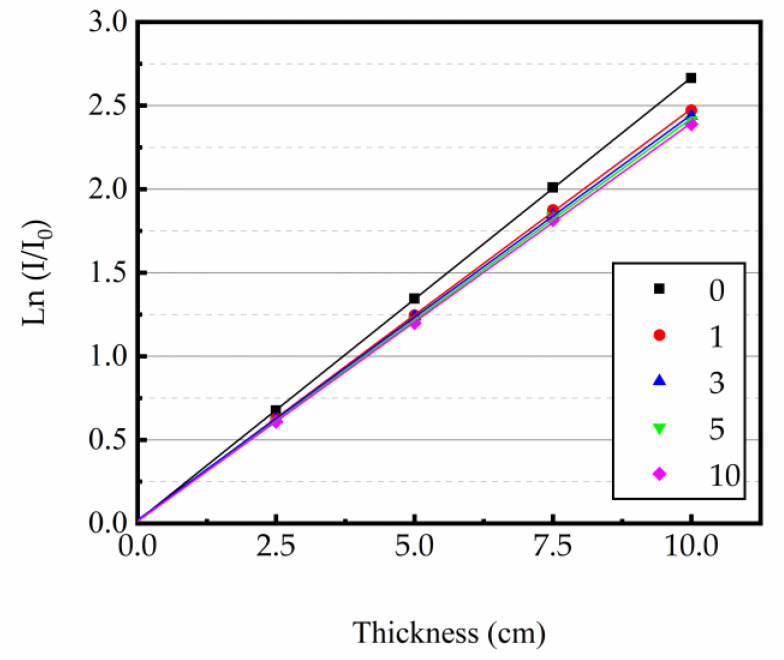
Relationship between ln(I/I_0_) and concrete thickness at 300 °C.

**Figure 11:**
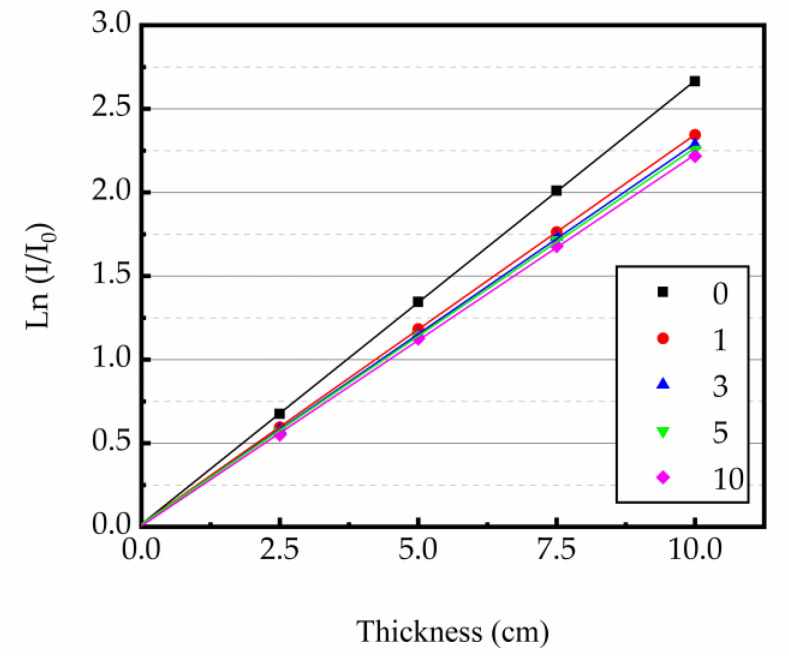
Relationship between ln(I/I_0_) and concrete thickness at 400 °C.

At 0.662 MeV energy level, the linear attenuation coefficient (μ) was derived as the slope of the regression line from the thickness of concrete specimen and ln(I/I_0_). The relationship curves between the test sample thickness and ln(I/I_0_) are shown in Figure 9, Figure 10 and Figure 11. The test results indicated that the μ was reduced with the increase in heating times. The higher the heating temperature, the smaller the μ of the test sample, and the bigger the rate of change between different heating times. The results of gamma ray shielding tests of specimen under different heating times at different temperatures are shown in Table 5. When the heating temperature was 200 °C and the number of heating times was 10, the μ of the specimen decreased by 3.71% and the HVL, TVL, and Mfp increased by 3.85% compared with normal temperature; the μ changed by 1.46% and the HVL, TVL, and Mfp changed by 1.48%, compared to the specimen after the first heating. At the heating temperature of 300 °C and the number of heating times up to 10, the μ of the specimens reduced by 10.43%, and the HVL, TVL, and Mfp improved by 11.52% compared with normal temperature. The μ changed by 3.40% and the HVL, TVL, and Mfp changed by 3.52%, compared to the specimens after the first heating. At the heating temperature of 400 °C and the number of heating times was 10, the μ of the specimen decreased by 16.78%, and the HVL, TVL, and Mfp increased by 20.17% compared with normal temperature. The μ changed by 5.43% and the HVL, TVL, and Mfp changed by 5.74%, compared to the specimen after the first heating. The results show that the HVL, TVL, and Mfp were continuously changing with the heating process. In addition, the higher the temperature, the larger the HVL, TVL, and Mfp, and the larger the amount of variation between different heating times.

At a certain photon energy, the concrete with more mass in the unit volume has better gamma ray shielding performance [38,51]. Aggregate is most of the mass of concrete in heavy concrete. Therefore, the element components, density, and thermal stability of aggregate played a significant factor for the shielding performance of concrete at high temperature. For magnetite concrete, it was relatively small (mainly the evaporation of water and the decomposition of partial hydration) under repeated heating at 200, 300, and 400 °C. This aggregate was relatively stable at high temperature, which means that the number of heavy elements in concrete did not decrease. Therefore, the change of concrete density may not be the main reason for the change of shielding performance of concrete. It has been shown that the increase of crack width in concrete will reduce its shielding performance [52]. To be specific, the increase in the porosity of concrete caused by evaporated water (pore water and hydration product crystal water) and the cracks within the concrete caused by repeated heating at high temperature mainly due to the decrease in the gamma ray linear attenuation coefficient.

#### 3.4.2. Gamma Rays Transmission Rate

**Figure 12:**
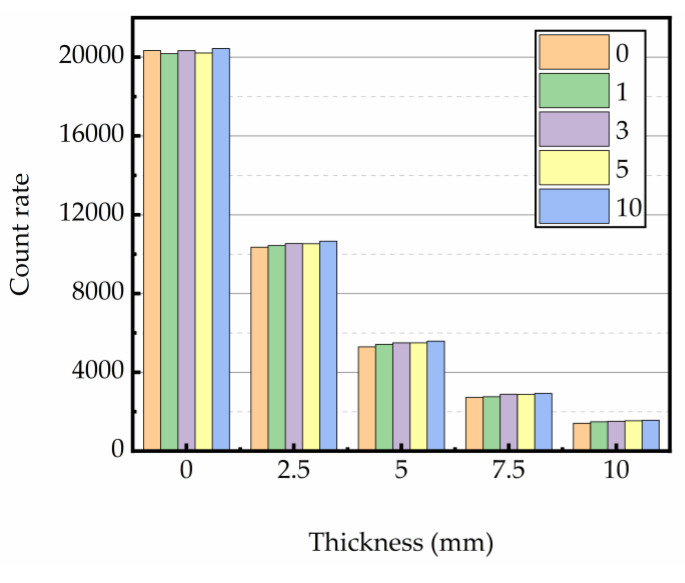
Relationship between thickness and counts at 200 °C.

**Figure 13:**
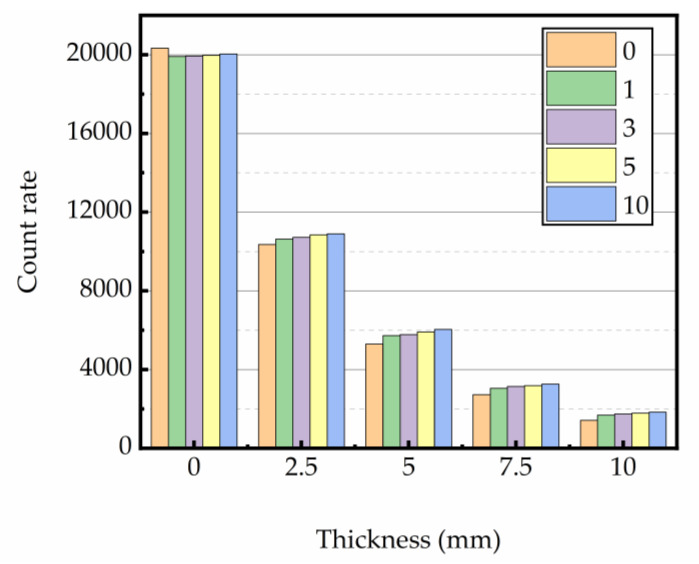
Relationship between thickness and counts at 300 °C.

**Figure 14:**
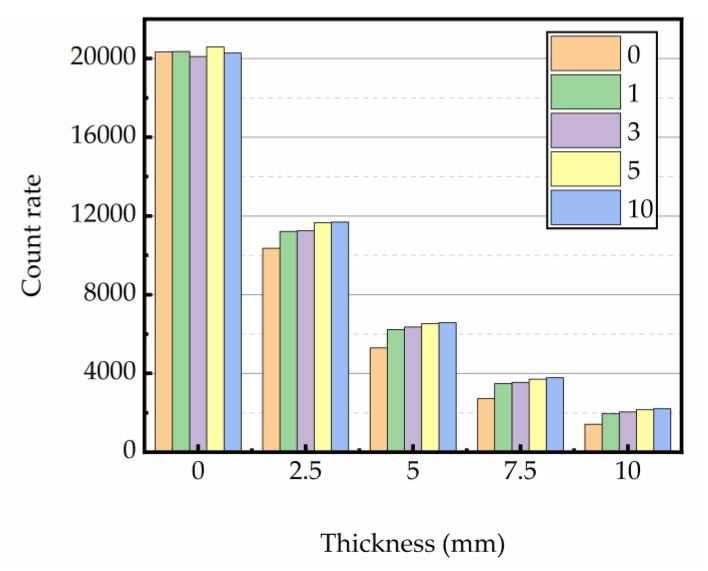
Relationship between thickness and counts at 400 °C.

**Figure 15:**
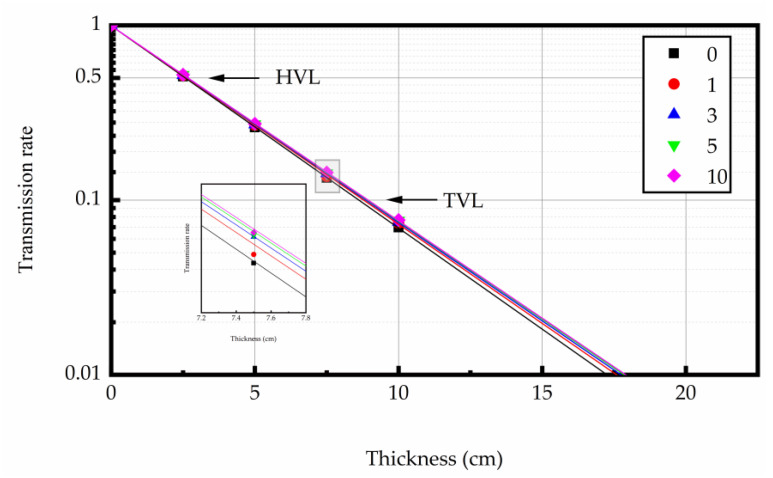
The relation between gamma-ray transmission rate and the concrete thickness at 200 °C.

**Figure 16:**
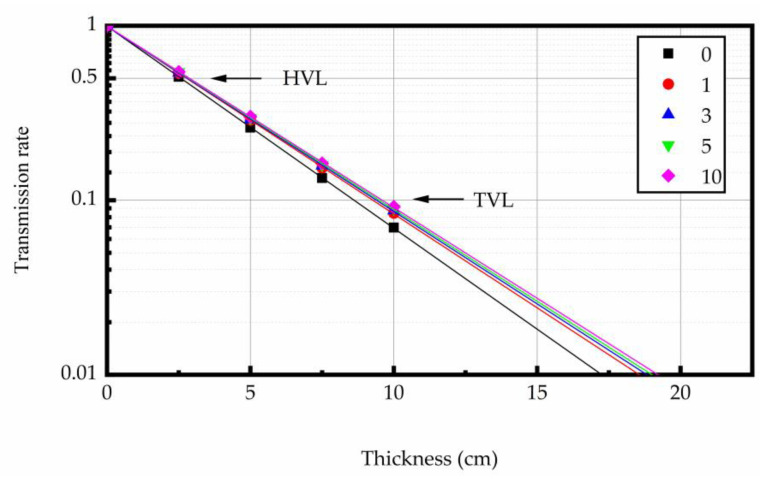
The relation between gamma-ray transmission rate and the concrete thickness at 300 °C.

**Figure 17:**
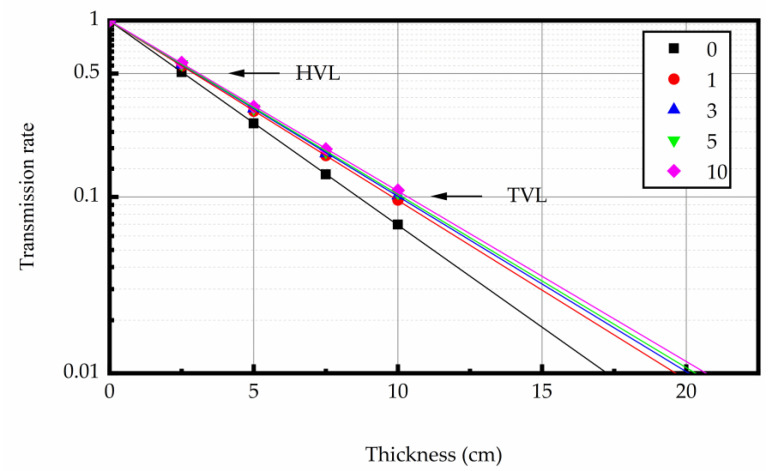
The relation between gamma-ray transmission rate and the concrete thickness at 400 °C.

The relation between gamma ray intensity (net of background radiation) and sample thickness is shown in Figure 12, Figure 13 and Figure 14. The test data indicated that gamma ray intensity reduced as the increasing thickness of magnetite concrete specimens. When the thickness of magnetite specimen was the same, the gamma ray intensity had a certain variation with temperature; the variation of gamma ray intensity with the number of heating was small. The relationship between gamma-ray transmission rate and thickness of the sample is shown in Figure 15, Figure 16 and Figure 17. The results showed that the minimum shield thickness in magnetite concrete increases with the increase of heating number and heating temperature. When the gamma-ray transmission rate is the same, the minimum shielding thickness of the test sample is inversely related to the heating temperature and the number of heating. The variation of the minimum attenuation layer thickness of the specimen with temperature and number of heating was the same as that of the HVL, TVL, and Mfp. In conclusion, the gamma shielding capacity of the specimens all decreased with the increase in heating times and heating temperature, and the higher the heating temperature, the greater the change brought about by the increase in heating times. Due to the specificity of radiation shielding concrete application, we should pay attention to the effect of environmental temperature changes in the performance of the shielding layers. Additionally, from the perspective of safety and economy, we should choose the best shielding thickness for the actual project’s building structure design.

## 4. Conclusions

The mass loss test, cracking tensile strength test, and gamma ray shielding test of magnetite concrete after repeated heating at 200, 300, and 400 °C are presented in this paper. The following conclusions can be drawn from the experimental results:

1. The changes in the apparent characteristics and mass loss of magnetite concrete varied with the increase in heating temperature. It was shown that the crack width, length, and number of specimens increased with the increase in heating times and heating temperature.
2. Magnetite concrete has good thermal stability, and its relative mass loss varies less with the number of heating times and temperature. The relative mass loss was only 2.67%, 2.93%, and 3.59% after heating the specimens at 200, 300, and 400 °C 10 times. The higher the heating temperature, the greater the relative mass loss of the samples, and the final relative mass loss of the samples tended to be constant for each heating temperature when the concrete was left more intact.
3. The splitting tensile strength of magnetite concrete decreased as there was an increase in heating times and heating temperature. Additionally, the higher the heating temperature, the greater the variation in the splitting tensile strength of concrete, and the more serious the deterioration of mechanical performance of concrete. The greatest change in splitting strength of magnetite radiation shielding concrete was observed in the first heating. The relative splitting strength changes of the specimens under heating temperatures of 200, 300, and 400 °C were 19.99%, 31.23%, and 51.66%, respectively. As the heating temperature was 200, 300, and 400 °C, and heating times up to 10, the relative tensile strength reductions in the specimens compared to the first heating were 5.52%, 12.77%, and 18.84%.
4. The linear attenuation coefficient of magnetite concrete gradually decreased with the increase in heating times and heating temperature, and its HVL, TVL, and Mfp gradually increased. When the gamma ray transmission rates were the equal, the minimal shielding thickness of magnetite concrete gradually climbs with the increase in the heating number and the heating temperature. In addition, with the increase in heating temperature, the greater the rate of change between different heating times. Therefore, the deterioration of physical, mechanical, and gamma shielding properties of the concrete is more severe the higher the temperature to which the concrete is subjected and the more drastic the temperature change during the service life of the magnetite radiation shielding concrete.

## Figures and Tables

**Table 1:** Chemical composition of cement and aggregates (wt.%).

Material Type	CaO	SiO_2_	Al_2_O_3_	Fe_2_O_3_	Fe_3_O_4_	MgO	Na_2_O	K_2_O	SO_3_	LOI
Cement	62.05	22.51	4.33	3.30	-	1.49	0.96	1.28	2.02	2.06
Magnetite Aggregate	3.26	7.94	5.55	-	78.52	2.43	0.54	0.11	0.26	1.40

**Table 2:** Basic performance of magnetite aggregate.

Aggregates	Particle Size (mm)	Apparent Density (kg/m^3^)	Water Content (%)	Water Absorption (%)	Crush Index (%)
Fine	0–5	4545	0.1	0.7	-
Coarse	5–20	4415	0.1	0.3	7.1

**Table 3:** Mix ratio of magnetite concrete.

Concrete Type	Water Binder Rate	Mix Ratio (kg/m^3^)			
		Cement	Water	Fine Aggregates	Coarse Aggregates
MC	0.60	308.3	185.0	1263.4	1895.0

**Table 4:** Splitting tensile strength of magnetite concrete exposed to multiple heating at high temperature.

T (°C)	Times	f_t, T_ (Mpa)	T (°C)	Times	f_t, T_ (Mpa)	T (°C)	Times	f_t, T_ (Mpa)
200 °C	0	2.577	300 °C	0	2.577	400 °C	0	2.577
	1	2.062		1	1.772		1	1.246
	3	2.034		3	1.718		3	1.155
	5	1.994		5	1.569		5	0.877
	10	1.919		10	1.443		10	0.760

**Table 5:** Gamma-ray shielding experimental results of magnetite samples under repeated heating at high temperature.

T (°C)	Times	μ (cm^−1^)	HVL (cm)	TVL (cm)	Mfp (cm)	μ_m_ (g/cm^2^)	Relative Coefficient (%)
25 °C	0	0.266 ± 0.001	2.602 ± 0.008	8.643 ± 0.028	3.754 ± 0.012	0.074	100.000
200 °C	1	0.260 ± 0.001	2.663 ± 0.009	8.845 ± 0.028	3.841 ± 0.012	0.073	97.714
	3	0.259 ± 0.001	2.673 ± 0.009	8.878 ± 0.028	3.856 ± 0.012	0.073	97.354
	5	0.257 ± 0.001	2.695 ± 0.009	8.953 ± 0.029	3.888 ± 0.012	0.072	96.539
	10	0.257 ± 0.001	2.702 ± 0.009	8.976 ± 0.029	3.898 ± 0.013	0.072	96.287
300 °C	1	0.247 ± 0.001	2.803 ± 0.009	9.311 ± 0.030	4.044 ± 0.013	0.071	92.826
	3	0.244 ± 0.001	2.843 ± 0.009	9.445 ± 0.030	4.102 ± 0.013	0.071	91.512
	5	0.241 ± 0.001	2.873 ± 0.009	9.544 ± 0.030	4.145 ± 0.013	0.070	90.559
	10	0.239 ± 0.001	2.902 ± 0.009	9.639 ± 0.031	4.186 ± 0.013	0.070	89.671
400 °C	1	0.234 ± 0.001	2.957 ± 0.009	9.822 ± 0.031	4.266 ± 0.014	0.069	87.995
	3	0.228 ± 0.001	3.034 ± 0.010	10.079 ± 0.033	4.377 ± 0.014	0.068	85.752
	5	0.226 ± 0.001	3.073 ± 0.010	10.207 ± 0.033	4.433 ± 0.015	0.068	84.678
	10	0.222 ± 0.001	3.127 ± 0.010	10.386 ± 0.033	4.511 ± 0.014	0.067	83.219

These are the experimental results of a 10 cm thick concrete sample.

## Data Availability

Not applicable.
